# The experience of financial burden for patients with multimorbidity: A protocol for a systematic review of qualitative research

**DOI:** 10.12688/hrbopenres.12915.2

**Published:** 2020-03-26

**Authors:** James Larkin, Louise Foley, Susan M. Smith, Patricia Harrington, Barbara Clyne

**Affiliations:** 1 HRB Centre for Primary Care, Royal College of Surgeons in Ireland, Dublin, Ireland; 2 School of Psychology, National University of Ireland Galway, Galway, Ireland; 3 Health Information and Quality Authority, Dublin, Ireland

**Keywords:** Multimorbidity, costs, financial burden, qualitative systematic review, protocol

## Abstract

**Introduction:** Multimorbidity is increasingly important due to its high disease burden, prevalence and related high healthcare utilisation. For patients, there is also a high financial burden due to direct and indirect costs arising from their multimorbidity. It is unclear how this financial burden affects patients. This study aims to synthesise qualitative evidence exploring the experience of financial burden from the perspective of patients with multimorbidity.

**Methods:** The review will be reported using the ENTREQ guidelines. A systematic search of Lilacs, PubMed, CINAHL, EMBASE, PsycINFO, and Applied Social Sciences Index and Abstracts will be conducted using a predefined search strategy. A search of fourteen pre-specified websites will be conducted for grey literature. Forward and backward citation checking of included studies will be conducted also. Studies will be included if they contain primary qualitative research and reference the experience of financial burden from the perspective of adult (≥ 18 years) community dwelling patients with multimorbidity. Studies from any country and in any language will be included. Titles and abstracts of search results will be screened; if a study appears relevant, then full-texts will be screened for eligibility. Study characteristics of included articles will be extracted. Study quality will be evaluated using the critical appraisal skills programme (CASP) checklist for qualitative research. These three processes will be carried out by two reviewers independently. Thematic-synthesis will be used to analyse data. This will be carried out by one reviewer and cross-checked by a second reviewer. The GRADE CERQual approach will be used to assess the overall confidence in the evidence.

**Discussion: **This review will identify evidence on the experiences of financial burden for patients with multimorbidity and forms part of a project to support consideration of financial burden for patients in the development of clinical guidelines in Ireland.

**PROSPERO registration number:** CRD42019135284

## Introduction

Chronic disease has become one of the biggest challenges for healthcare systems globally ^1^. This has brought into focus the phenomenon of multimorbidity, the presence of two or more chronic diseases in a patient ^2^. Multimorbidity is of increasing concern due to the high disease burden and the related high rates of healthcare utilisation. The estimated prevalence of multimorbidity in the general population ranges from 13% to 72% ^2^. These variations are largely accounted for by differences in settings and age groups across prevalence studies. The prevalence is likely increasing due to the ageing of the population globally ^3^. Despite this, healthcare systems internationally are primarily single disease focused ^4^. This single disease focus is reflected in clinical guidelines, which primarily treat diseases in isolation and rarely account for patients with multimorbidity. This creates a significant treatment burden ^4^ which has several consequences for patients with multimorbidity, including a financial burden.

Financial burden refers to direct medical costs, direct non-medical costs and indirect-costs accruing to patients as a result of their multimorbidity. The financial burden of multimorbidity on patients is widespread and can be significant. A systematic review of cost-of-illness studies concluded that multimorbidity was always associated with higher out-of-pocket (OOP) expenditure than single or no chronic conditions ^5^. Another systematic review found that a greater number of conditions present in a person was associated with higher OOP expenditure on medications ^6^. This financial burden is of particular concern in terms of equity, as multimorbidity disproportionately affects patients from lower socioeconomic groups ^7^.

Much of this economic-burden associated with multimorbidity arises from OOP expenditure or direct medical costs but it may also arise from direct non-medical costs including transportation to healthcare appointments and indirect costs including work absences. The economic-burden associated with multimorbidity can have negative effects including reduced medication adherence primarily due to inability to purchase required medication ^6^, impoverishing spending (i.e., spending that pushes a household below an agreed poverty line) ^5^, and reduced quality-of-life ^5^.

Several qualitative studies have examined patients’ lived experience of multimorbidity ^8, 9^. Many of these studies have a brief focus on experience of financial burden. By synthesising these studies, a broader picture of this experience can be provided. It has been suggested that by synthesising many studies the patient is given a greater voice ^10^. The authors therefore sought to synthesise qualitative research exploring experience of financial burden for patients with multimorbidity.

## Research questions

What are the experiences of patients with multimorbidity of financial burden?

How does financial burden affect interactions between patients with multimorbidity and the healthcare system?

How does financial burden impact on treatment burden for patients with multimorbidity?

## Methods

### Design

There are recognised challenges in upholding the complexity and context of primary qualitative research when conducting a qualitative systematic review. However, patients’ views and experiences should inform decision making ^11^ and these can be ascertained using qualitative methods ^11^. By providing a systematic review and synthesis of this research, policy-makers can be more comprehensively informed ^11^.

This review will be conducted and reported using the ENTREQ guidelines ^12^. The review protocol is written in accordance with the PRISMA-P guidelines (reporting guidelines ^13^).

### Search strategy

The following databases will be searched: Lilacs, PubMed, CINAHL, EMBASE, PsycINFO, and Applied Social Sciences Index and Abstracts. Additionally, forward and backward citation checking of included studies will be conducted. Content experts will be contacted requesting information on any articles the content experts feel are relevant. For the grey-literature search a list of websites considered relevant by the research team were chosen (extended data ^13^). Databases will be searched from inception using combinations of Mesh terms and key-words (extended data ^13^).

The search strategy was developed in conjunction with a librarian. Scoping searches were conducted using key words related to financial burden and qualitative research. The terms for multimorbidity were taken from the Cochrane systematic review examining multimorbidity interventions ^13^. The search strategy presented in extended data ^13^ is based on Medline and will be tested and adapted for all other databases.

### Screening

Search results will be exported to Endnote X8, duplicate entries removed and then imported into Covidence. For step one, titles will be screened by a single reviewer (JL) to remove entries that are clearly unrelated to the research question. For step two, two reviewers (JL, LF) will screen titles and abstracts independently; according to the inclusion criteria ( Table 1). Any disagreements will be resolved through discussion. If this does not lead to agreement, then a third reviewer will decide on inclusion for full-text review. Potentially eligible full texts will be independently evaluated by two reviewers (JL, LF) against the inclusion and exclusion criteria and disagreement will be managed by a third reviewer (SS).

**Table 1.** Inclusion and exclusion criteria based on modified PICoS ^15^.

PICoS	Inclusion Criteria	Exclusion Criteria
**Population**	Identified as patient with multimorbidity ≥ 2 chronic diseases Community dwelling adults (≥ 18 years old)	Single condition focus
**Phenomenon of Interest**	Financial burden for patients	
**Context**	Any country Primary and secondary care	Residential healthcare facilities
**Study Type**	Qualitative Original research (e.g., interviews or focus groups) Mixed methods	Quantitative

### Eligibility criteria

Only studies using a qualitative design, with primary data collection, referencing experiences of financial burden, and examining community-dwelling adults (≥ 18 years) with multimorbidity will be included ( Table 1). Studies examining patients with non-specific chronic disease will be included if they include patients with multimorbidity and do not have a single condition focus. Qualitative design refers to studies which use a method of data collection and data analysis which are recognised qualitative methods ^14^, for example interviews, focus groups, thematic analysis, and content analysis. Financial burden refers to the direct medical costs, direct non-medical costs and indirect costs experienced by patients. It is expected that the focus of studies will not exclusively be financial burden. Therefore, papers with broader focuses, such as the experience of multimorbidity, will be reviewed for inclusion. Also, many studies concerning financial burden and multimorbidity relate to polypharmacy, therefore studies concerning the experience of polypharmacy for patients with multimorbidity will be included if they meet the inclusion criteria. Studies without reference to financial burden will be excluded. Only data related to experience of financial burden for patients with multimorbidity will be included in the analysis. Mixed-methods studies with primary qualitative data collection will be included if they meet the inclusion criteria and where it is possible to extract the findings derived from the qualitative research. No language restrictions will apply.

### Data extraction and analysis

Two reviewers will extract study characteristics independently using a proforma (see extended data ^13^) under the following headings: setting, country, year of publication, methodology, participants (age, gender, socioeconomic status, conditions), sampling strategy, data-analysis technique, and definition of financial burden. Conflicts will be resolved by a third reviewer (BC).

Data (quotes, themes and author interpretations) will be extracted verbatim from the results section of included studies. This process will be conducted by a single reviewer (JL), and then cross-checked by a second reviewer (LF) to increase confirmability. Only data considered relevant to the research questions will be extracted. If information is unavailable from the full-text, then the corresponding author will be contacted for clarification. If there is no reply, then a follow-up email will be sent one week later and if no reply is received within one week of the second email then a decision will be made on inclusion based on information available.

Thematic-synthesis, as described by Thomas and Harden ^16^, will be used. Thematic-synthesis is an inductive approach which is often used for studies with ‘thin’ data and analysis ^16^. It is also used to draw inference based on common themes from studies with different designs and perspectives ^17^. Thematic-synthesis consists of a three step process; step one consists of line-by-line coding of the data of included studies. The second step involves organisation or grouping these codes into related areas to construct ‘descriptive’ themes. In step three, the descriptive themes will be iteratively examined and compared to refine the relationship between them and generate analytical themes that is, themes that go beyond the descriptive themes to provide new insights related to the review question. Data will be coded using NVIVO version 12. Following multiple readings of the included papers data-analysis will be carried out by a single reviewer (JL) following the three steps outlined above. In order to increase confirmability of the analysis, all studies will be independently read by a second reviewer (LF) to crosscheck the coding structure and themes developed. This process will be overseen by a third reviewer (BC). In order to increase the credibility of the findings, an overview of the results will be brought for discussion to a panel of public and patient representatives with experience of multimorbidity. Direct quotations from study participants will be presented in italics to distinguish them from second order data (author interpretations).

### Quality-appraisal of included studies

The critical appraisal skills programme (CASP) checklist for qualitative research ^18^ will be used to assess the methodological quality of all included studies. Two reviewers (JL and LF) will independently evaluate each study and any differences will be resolved through discussion. If this does not lead to agreement, then a third reviewer (BC) will adjudicate. Studies will not be excluded based on quality-appraisal. Quality-appraisal will be used as a means of discussing the quality of the included studies and to inform the GRADE CERQual (Confidence in the Evidence from Reviews of Qualitative research) assessment of confidence in the review findings ^19^.

### Assessing the quality of the body of evidence

The review is intended to form part of a project which will inform how the specific needs of patients with multimorbidity are considered within clinical guidelines in Ireland. Therefore, the GRADE CERQual approach will be used to summarise our confidence in the evidence ^19^. Four components contribute to an assessment of confidence in the evidence for an individual review finding: methodological limitations, relevance, coherence, and adequacy of data. Methodological limitations assesses the conduct and design of the primary studies in relation to the findings to which they are contributing. Relevance assesses the degree to which the evidence from the primary studies applies to the context of the review question. Coherence assesses how well the findings are supported by and fit with the primary studies. Adequacy of data assesses how much data supports a finding and how rich this data is. Confidence in the evidence will be graded as high, moderate, low, or very low. This assessment will also be conducted in duplicate (JL and LF) and discussed amongst the research team.

***Reflexivity***. It is important to consider all findings in the context of research team members’ personal worldviews and experiences. Three authors have a background in social science; in psychology (JL, LF) and sociology and health services research (BC). One author has a background in general practice (SS) and is a leading expert in multimorbidity research, and one has a background in pharmacy and health economics (PH). The authors have operated within the Irish and other healthcare contexts. In relation to analysis, the lead researcher conducting the analysis (JL) does not have any chronic conditions. The authors will examine and discuss their preconceptions and beliefs surrounding the research questions, and consider the relevance of these preconceptions during each stage of analysis.

***Dissemination of information***. The review will be published in a peer-reviewed journal, reported using the ENTREQ guidelines ^12^. The review will also be presented at a relevant conference and disseminated to policy-makers, patients, and the public.

***Study status***. At time of publication the study is ongoing, and title and abstract screening is underway.

## Discussion

The review will add to the knowledge base of how financial burden affects patients with multimorbidity as well as informing potential policy and practice interventions for patients with multimorbidity. This review also forms part of a project which, as a whole, will contribute to developing guidance of how the specific needs of patients with multimorbidity are considered within clinical guidelines in Ireland, and internationally. The review will inform the development of a national survey that will quantify economic burden for patients with multimorbidity in Ireland. Limitations include the potential paucity of data in included studies.

## Data availability

### Underlying data

No data are associated with this article

### Extended data

Open Science Framework: The experience of financial burden for patients with multimorbidity: A protocol for a systematic review of qualitative research: Extended data. https://doi.org/10.17605/OSF.IO/PN42R ^13^

This project contains the following extended data:

- Proforma.docx (a proforma with all headings under which study characteristics will be extracted)
- Medline (OVID) Search strategy.docx (The mix of key words and mesh terms that will be used to search Medline and that will be transferred to other databases for searchers)
- Grey literature search.docx (the list of websites that will be searched for grey literature using a variation of the Medline search strategy)

### Reporting guidelines

PRISMA-P checklist for ‘The experience of financial burden for patients with multimorbidity: A protocol for a systematic review of qualitative research’, https://doi.org/10.17605/OSF.IO/PN42R ^18^

Data are available under the terms of the Creative Commons Zero “No rights reserved” data waiver (CC0 1.0 Public domain dedication).
