# Supplementary material for: The experience of financial burden for patients with multimorbidity: A protocol for a systematic review of qualitative research
Source: HRB Open Res. 2020 Mar 26;2:16. Originally published 2019 Jul 25. [Version 2] doi: 10.12688/hrbopenres.12915.2 (PMC7309055; doi:10.12688/hrbopenres.12915.2)
Supplement: Peer review history

# Peer review: Reviewer response for version 2

**DOI:** 10.21956/hrbopenres.14115.r27266

**Published:** 2020-06-18

**Authors:** Carole Cummins

**Affiliations:** 1 Institute of Applied Health Research, College of Medical and Dental Sciences, Murray Learning Centre, University of Birmingham, Birmingham, UK

The authors have addressed my previous comments, and I have no further improvements to suggest or clarifications to suggest.

Is the study design appropriate for the research question?

Yes

Is the rationale for, and objectives of, the study clearly described?

Yes

Are sufficient details of the methods provided to allow replication by others?

Yes

Are the datasets clearly presented in a useable and accessible format?

Not applicable

Reviewer Expertise:

Research relevant to this review: Systematic review including metasynthesis, chronic and medically complex conditions in childhood.

I confirm that I have read this submission and believe that I have an appropriate level of expertise to confirm that it is of an acceptable scientific standard.

# Peer review: Reviewer response for version 1

**DOI:** 10.21956/hrbopenres.13993.r27148

**Published:** 2020-02-26

**Authors:** Carole Cummins

**Affiliations:** 1 Institute of Applied Health Research, College of Medical and Dental Sciences, Murray Learning Centre, University of Birmingham, Birmingham, UK

*Is the rationale for, and objectives of, the study clearly described?*

 Yes.

 The rationale for the study is described. Something the authors may wish to consider is that the scope of the review is global, but the aim is to inform Irish guidelines: the review will include research from both Upper Income Country and Low and Middle Income Country settings. While the ambitious scope may potentially add value to the review, the authors could discuss how they plan to take account or acknowledge differences in setting, health care systems and access to healthcare.

*Is the study design appropriate for the research question?*

 Yes.

 This report is a protocol for a systematic review of qualitative research providing data on the experience of financial burden of patients with multiple morbidity. A completed PRISMA-P checklist for a systematic review protocol is attached and appropriately completed. There is a PROSPERO database registration which should facilitate publication of the completed review.

 The methods are well described and appropriate but I do have a few questions and in places perhaps there could be further explanation/exposition.

 A proposed search is clearly described in the supplementary materials but, given the broad scope of multimorbidity and its contexts, it would have been good to see more about how this search strategy was developed. Were scoping strategies used? Also, will the validity of the search be considered and if necessary adapted? Will the example search for Medline be tested and if necessary adapted for other databases?

 A minor point is that a little more detail could be given on the inclusion criteria at the full text stage. I assume that all citations selected on the basis of title and abstract will undergo full text screening by two reviewers, with resolution by a third, this is not precisely the same as the citation screening process where one reviewer removes clearly irrelevant citations. A sentence explicitly stating the full text inclusion process would be helpful.

 The suggested thematic analysis is appropriate, as is the process for generating codes and themes with validation from a second reviewer. Will first order data (direct quotations etc) be distinguished from second order data (author's interpretations) in reporting of the results? As you will potentially be including data from UMCs and LMICs and from settings with health systems that may differ markedly regarding co-payments and access to healthcare, how will you deal with these differences in the analysis. You might, for example, want to consider a thematic framework approach.

Is the study design appropriate for the research question?

Yes

Is the rationale for, and objectives of, the study clearly described?

Yes

Are sufficient details of the methods provided to allow replication by others?

Yes

Are the datasets clearly presented in a useable and accessible format?

Not applicable

Reviewer Expertise:

Research relevant to this review: Systematic review including metasynthesis, chronic and medically complex conditions in childhood.

I confirm that I have read this submission and believe that I have an appropriate level of expertise to confirm that it is of an acceptable scientific standard, however I have significant reservations, as outlined above.

# Response

**Published:** 2020-03-16

**Authors:** James Larkin

**Affiliations:** Royal College of Surgeons in Ireland, Ireland

Dear Dr. Cummins,

Thank you for reviewing our submission and for your thoughtful feedback. Below are our responses. Text labelled ‘reviewer’ are the comments of the reviewer. Text labelled ‘authors’ is the response of the authors.

**Reviewer**: While the ambitious scope may potentially add value to the review, the authors could discuss how they plan to take account or acknowledge differences in setting, health care systems and access to healthcare.

**Authors: **Indeed, we anticipate there will be much variation in relation to setting and access in the included studies, which will have an impact on financial burden. We are using the GRADE CERQual tool to assess confidence in the review findings. This approach will allow us to take into account the impact of variation. Specifically, the ‘relevance’ section of the CERQual tool will incorporate an assessment of the extent to which the body of data from included studies is applicable to the context of the research question. To address this we have added sentences to clarify what each of the four domains assess (amended paragraph under ‘Assessing the quality of the body of evidence’).

Also, the table of included studies (which will be included as an appendix in the final publication) will include the country in which the study was carried out. This can be used for reference. In addition, the study characteristics, which will be described in the results, will outline the main countries in which the included studies were carried out.

**Reviewer**: It would have been good to see more about how this search strategy was developed. Were scoping strategies used? Also, will the validity of the search be considered and if necessary adapted? Will the example search for Medline be tested and if necessary adapted for other databases?

**Authors**: There was work done on this that we had not included. Thank you for drawing our attention to this. A paragraph (paragraph two under ‘Search strategy’) has been added to address the issues you have raised and provide more detail on the scoping search.

**Reviewer**: A sentence explicitly stating the full text inclusion process would be helpful.

**Authors**: Indeed, this was not clear from the original text. A sentence has been added (last sentence of paragraph one under ‘screening’)  to clarify which processes will be used for full text inclusion. 

**Reviewer**: Will first order data (direct quotations etc) be distinguished from second order data (author's interpretations) in reporting of the results?

**Authors**: First order data (direct quotations etc.) will be distinguished from second order data (author's interpretations) in reporting of the results. This will be done through the use italics for first order data. This has been made clear in the updated protocol (a new sentence has been added at the end of paragraph three under ‘data extraction and analysis’).

**Reviewer**: As you will potentially be including data from UMCs and LMICs and from settings with health systems that may differ markedly regarding co-payments and access to healthcare, how will you deal with these differences in the analysis. You might, for example, want to consider a thematic framework approach.

**Authors**: Dealing with the differences between countries does present a challenge. There are two areas within the CERQual tool, which will facilitate this: coherence and relevance. To clarify this we have added sentences to clarify what each of the four domains assess (amended paragraph under ‘Assessing the quality of the body of evidence’, added after sentence three).

In addition, within the analysis, if there are areas that are likely to be particularly effected by the health system then the countries in which the theme is applicable will be mentioned.

# Peer review: Reviewer response for version 1

**DOI:** 10.21956/hrbopenres.13993.r26718

**Published:** 2019-08-01

**Authors:** Katie I. Gallacher

**Affiliations:** 1 General Practice and Primary Care, Institute of Health and Wellbeing, College of Medical, Veterinary and Life Sciences, University of Glasgow, Glasgow, UK

This is an interesting and much needed systematic review protocol. The aim is to synthesize the qualitative evidence relating to the experience of financial burden for people with multimorbidity. I have a few comments.

**Introduction**:

1. The authors state that variations in estimated prevalence of multimorbidity are due to differences in settings and age groups. This is true but it is also due to differences in methods of measurement.
2. Clinical guidelines are largely disease focussed but it would be worth mentioning the NICE multimorbidity guidelines 2016 ( https://www.nice.org.uk/guidance/ng56) which mentions treatment burden.
3. There are several groups who have explored treatment burden in multimorbidity and it would be worth citing these:
  Eton DT, Ridgeway JL, Egginton JS, Tiedje K, Linzer M, Boehm DH *et al*.: **Finalizing a measurement framework for the burden of treatment in complex patients with chronic conditions.** *Patient Relat Outcome Meas* 2015, **6:** 117-126. ^1^
  Tran VT, Barnes C, Montori VM, Falissard B, Ravaud P: **Taxonomy of the burden of treatment: a multi-country web-based qualitative study of patients with chronic conditions.** *BMC Med* 2015, **13:** 115. ^2^
  Sav A, Sav A, endall E, cMillan SS, elly F, hitty JA *et al*.: **'You say treatment, I say hard work': treatment burden among people with chronic illness and their carers in Australia.** *Health Soc Care Community* 2013. ^3^

**Methods:**

1. Have the authors considered publishing the protocol on PROSPERO?
2. Using one reviewer for initial exclusion during title screening should be acknowledged as a limitation, as this risks bias. One option would be to allow one reviewer to include studies at this stage but ideally two should be involved in exclusion.
3. Will postal questionnaires be excluded? This should be added to the criteria.
4. There is a potential for a large amount of papers to be full paper screened and included due to the fact that information on financial burden may be 'hidden' in studies with other objectives e.g. those that aim to explore the experience of multimorbidity more generally. It may be useful to have a clear cut off for inclusion e.g. if there is one item of data on financial burden in a paper and the rest is irrelevant, will this paper be included?
5. The methods for data analysis and quality appraisal appear very robust. 

Is the study design appropriate for the research question?

Yes

Is the rationale for, and objectives of, the study clearly described?

Yes

Are sufficient details of the methods provided to allow replication by others?

Yes

Are the datasets clearly presented in a useable and accessible format?

Not applicable

Reviewer Expertise:

Treatment burden, multimorbidity, stroke.

I confirm that I have read this submission and believe that I have an appropriate level of expertise to confirm that it is of an acceptable scientific standard.

## References

[1] : Finalizing a measurement framework for the burden of treatment in complex patients with chronic conditions. *Patient Relat Outcome Meas*.2015;6: 10.2147/PROM.S78955 117-26 10.2147/PROM.S78955 25848328PMC4383147

[2] : Taxonomy of the burden of treatment: a multi-country web-based qualitative study of patients with chronic conditions. *BMC Med*.2015;13: 10.1186/s12916-015-0356-x 115 10.1186/s12916-015-0356-x 25971838PMC4446135

[3] : 'You say treatment, I say hard work': treatment burden among people with chronic illness and their carers in Australia. *Health Soc Care Community*.2013;21(6) : 10.1111/hsc.12052 665-74 10.1111/hsc.12052 23701664
